# NDRG2 gene expression pattern in ovarian cancer and its specific roles in inhibiting cancer cell proliferation and suppressing cancer cell apoptosis

**DOI:** 10.1186/s13048-020-00649-0

**Published:** 2020-04-28

**Authors:** Fenhong Kang, Yanlong Wang, Yaping Luo, Yongjun Zhang

**Affiliations:** 1 Department of Gynecology, Women and Children’s Hospital, School of Medicine, Xiamen University, Xiamen, 361001 Fujian China; 2 Department of Pathology, ZhongShan Hospital, Xiamen University, Xiamen, 361004 Fujian China

**Keywords:** Ovarian cancer, N-myc downstream-regulated gene 2 (NDRG2), Cell viability, Apoptosis, Cisplatin (DDP)

## Abstract

**Background:**

The cancer cell metastasis and the acquisition of chemotherapy resistance remain huge challenge for ovarian cancer treatment. Previously, N-myc downstream-regulated gene 2 (NDRG2) serves as a tumor suppressor for many cancers. Here, we attempted to investigate the specific roles of NDRG2 in ovarian cancer.

**Methods:**

The expression levels of NDRG2 were detected by qRT-PCR or Immunoblotting. CCK-8 assay was employed to examine the cell viability of ovarian cancer cells. The colony formation ability was determined by colony formation assay. Flow cytometry analyses were performed to detect the cell apoptosis and cell cycle. Xenograft tumor assay was performed to detect the in vivo function of NDRG2.

**Results:**

We revealed that NDRG2 mRNA expression and protein levels were downregulated within both ovarian cancer tissues and cell lines. The overexpression of NDRG2 dramatically inhibited the cell viability and colony formation and tumor growth, whereas promoted the cell apoptosis, cell cycle arrest in G1 phase within ovarian cancer cells. More importantly, NDRG2 overexpression significantly enhanced the suppressive roles of cisplatin (DDP) in ovarian cancer cell viability. On the contrary, NDRG2 silence exerted opposing effects on ovarian cancer cells.

**Conclusions:**

In summary, we provide a solid experimental basis demonstrating the tumor-suppressive effects of NDRG2 in inhibiting the cell proliferation, enhancing the cell apoptosis, eliciting the cell cycle arrest in G1 phase, and promoting the suppressive effects of DDP on the viability of ovarian cancer cells. NDRG2 administration presents a potent adjuvant treatment for ovarian cancer therapy.

## Introduction

Ovarian cancer is one of the deadliest malignancies in female [1, 2]. Since the incipient symptoms of ovarian cancer are obscure, most patients received diagnosis until entering the advanced stage [3–5]. Surgical treatment and platinum-based chemotherapy are major therapeutic strategies for ovarian cancers [6]. Unfortunately, these therapeutic methods seem to become less effective with the progression of the cancer. Moreover, the morbidity of the ovarian cancer also remains a higher level due to lack of reliable predictive biomarker, ovarian cancer cells metastasis and the resistance to chemotherapy [7]. Thus, it’s necessary to determine ovarian cancer pathophysiology and find new treatment methods.

Interestingly, it has been revealed the correlations between cancer (such as lung, prostate, liver, colorectal and breast cancer) and N-myc downstream-regulated gene 2 (NDRG2) [8–10]. NDRG2 is considered to be a tumor suppressor which contributes to not only hormone, ion, fluid metabolism and other cellular metabolic processes [11, 12], but also stress responses, like those under hypoxic environments and lipid toxicity [13, 14]. It has been demonstrated the correlations between NDRG2 and cancer within neurotumors [15, 16], gastroenteric tumors [9, 17], genitourinary tumors [18, 19], breast carcinoma [20, 21], lung carcinoma [10, 22], thyroid carcinoma [23], oral squamous-cell cancer [24], myeloid leukemia [25], and cervical cancer [14]. Collectively, the expression of NDRG2 is reduced within human tumors, while its overexpression suppresses the capacity of cancer cells to proliferate, migrate, metabolize and invade [26]; NDRG2 expression levels are negatively correlated with human cancer clinical and pathological conditions [26]. Nevertheless, little is known about the specific role of NDRG2 within ovarian cancer.

Herein, NDRG2 mRNA and protein expression showed to be monitored within ovarian cancer tissues and cells. Next, NDRG2 overexpression and silence were conducted in three cell lines of ovarian cancer; the specific effects of NDRG2 upon the viability, colony formation ability, apoptosis, cell cycle, and the sensitivity to cisplatin (DDP) treatment of ovarian cancer cells were evaluated. The NDRG2 inhibition capacity towards holding the growth and tumorigenesis of ovarian cancer was determined in vivo. In summary, we attempt to provide a solid experimental basis for understanding the cellular functions of NDRG2 on ovarian cancer cells.

## Materials and methods

### Clinical tissue samples

We collected a total of 6 paired non-cancerous (NC) and ovarian cancer tissues from patients received resection surgeries in Zhongshan hospital with the signed the consent from each patient. All the experiments in the present study were conducted with the approval of the Ethics Committee of Zhongshan hospital. The pathologic type of all samples was confirmed by two independent pathological experts. Tissues were frozen at − 80 °C immediately after sampling until further use.

### Cell lines and cell culture

A normal cell line, human ovarian surface epithelial cell line (HOSE, also known as HOSEpiC), was purchased from ScienCell (Cat. #7310; Carlsbad, CA, USA) and cultured in Ovarian Epithelial Cell Medium (OEpiCM, Cat. #7311; ScienCell). Ovarian cancer SKOV3 (ATCC® HTB-77™), OVCAR-3 (ATCC® HTB-161™), and CAOV3 (ATCC® HTB-75™) cell lines were obtained from ATCC (Manassas, VA, USA). SKOV3 cells were cultured in McCoy’s 5a Medium Modified (Catalog No. 30–2007; ATCC). CAOV3 cells were cultured in Dulbecco’s Modified Eagle’s Medium (Catalog No. 30–2002; ATCC). OVCAR-3 cells were cultured in RPMI-1640 Medium (Catalog No. 30–2001; ATCC). All the cells were cultured with 10% FBS (Invitrogen, Carlsbad, CA, USA) at 37 °C in 5% CO_2_.

Cells were transfected with scramble siRNA (negative control, si-NC; RiboBio, Guangzhou, China) or NDRG2 siRNA (si-NDRG2; RiboBio) with the help of Lipofectamine 3000 reagent (Thermo Fisher Scientific, Waltham, MA, USA). Cells were collected and used for further experiments 48 h after transfection.

### PCR-based analyses

Total RNA was extracted from target cells with the help of TRIzol reagent (Invitrogen). The reverse transcription of extracted RNAs into cDNA was performed with the help of Maxima First Strand cDNA Synthesis Kits (K1672; Thermo Fisher). The expression of mRNA was determined with an SYBR® Green Real-time PCR Master Mix (Sigma, St. Louis, MO, USA) using GAPDH as an endogenous control. All the results were processed and analyzed using the 2^-ΔΔCt^ method.

### Immunoblotting

Cell lysate was prepared using RIPA lysis buffer (Applygen, Beijing, China) and proteins were extracted. SDS-PAGE (10%) was used to separate the extracted proteins. After that, proteins were transferred onto PVDF membranes. Nonspecific antigen was blocked by 5% non-fat milk solution, and the membranes were washed three times using PBST. The membranes were then incubated overnight at 4 °C with anti-NDRG2 (Cat# 12015–1-AP, Proteintech, Rosemont, IL, USA), anti-BAX antibody (1:1000, Cat# ab32503), anti-BCL2 antibody (1:1000, Cat# ab32124), anti-Cleaved Caspase-3 antibody (Cat# ab2302), anti-Caspase-3 antibody (1:500, Cat# ab13847), anti-cyclin B1 (1:50000, Cat# ab32053), anti-cyclin A2 (1:2000, Cat# ab181591) (Abcam, Cambridge, MA, USA) and anti-β-actin (60008–1-Ig, Proteintech). After washing with PBST, the membranes were incubated with appropriate HRP goat anti-mouse/anti-rabbit IgG (Proteintech) at room temperature. The visualization of all the blots were conducted using enhanced chemiluminescence (ECL; Thermo Fisher).

### Cell viability determined by CCK-8 assay

A CCK-8 kit (Sigma-Aldrich, St. Louis, MO, USA) was employed to examine the cell viability of ovarian cancer cell lines in response to different treatment and/or transfection. Cells were placed into 96-well plates at a density of 1 × 10^4^ cells/well. CCK-8 solution was added at 0 h and 24 h thereafter and then cells were incubated for 4 h at 37 °C. The absorbance (OD value) was measured at 450 nm.

### Colony formation

The colony formation ability was determined. Cells were cultured 6-well plates at a density of 1 × 10^2^ cells/well. Fourteen days later, the colonies were fixed with methanol and stained with 0.1% crystal violet (Sigma-Aldrich). After that, the number of visible colonies were counted.

### Cell cycle and cell apoptosis determined by flow cytometry

Flow cytometry analyses were performed to detect the cell apoptosis and cell cycle. For cell apoptosis analysis, cells were collected, resuspended, and added with annexin V-FITC and PI. After 15 min of incubation, the cell apoptosis was analyzed.

For cell cycle analysis, cells were fixed with ethanol (70%, ice-cold) for 20 min and added with PI. After 20 min incubation, the cell cycle was analyzed.

### Xenograft mice assay in vivo

Pathogen free conditions were maintained through the lifetime of twelve male BALB/c nude mice (4 weeks old). The approval of xenograft in vivo assay was obtained from Zhongshan hospital. Firstly, overexpression of NDRG2 (Lv-NDRG2 OE) was performed using MISSION® shRNA lentiviral particles (Sigma-Aldrich), which were designed to overexpress the production of NDRG2 in SKOV3 cells. The cultures that were transfected with lentiviral particles with negative control vector were used as the control group (Lv-Vector). Subsequently, Lv-NDRG2 OE or Lv-Vector transfected SKOV3 cells (1 × 10^6^) were subcutaneous injected into the armpit of nude mice respectively. Caliper was adapted in measuring tumor volume following the length×width^2^/2 formula. The average volume of tumor was measured for 3 times every 3 days. At the termination of the experiment (the 25th day), mice were killed and the tumor was excised from each mouse to measure the average volume and weight.

### Data processing and statistical analysis

All data collected from three independent experiments were processed and analyzed by GraphPad (San Diego, CA, USA). The data was represented as mean ± SD. The comparison was conducted using paired or unpaired Student’s *t*-test. A *P* value of less than 0.05 was considered as statistically significant.

## Results

### The mRNA and protein expression of NDRG2 within tissues and cells

**Fig. 1:**
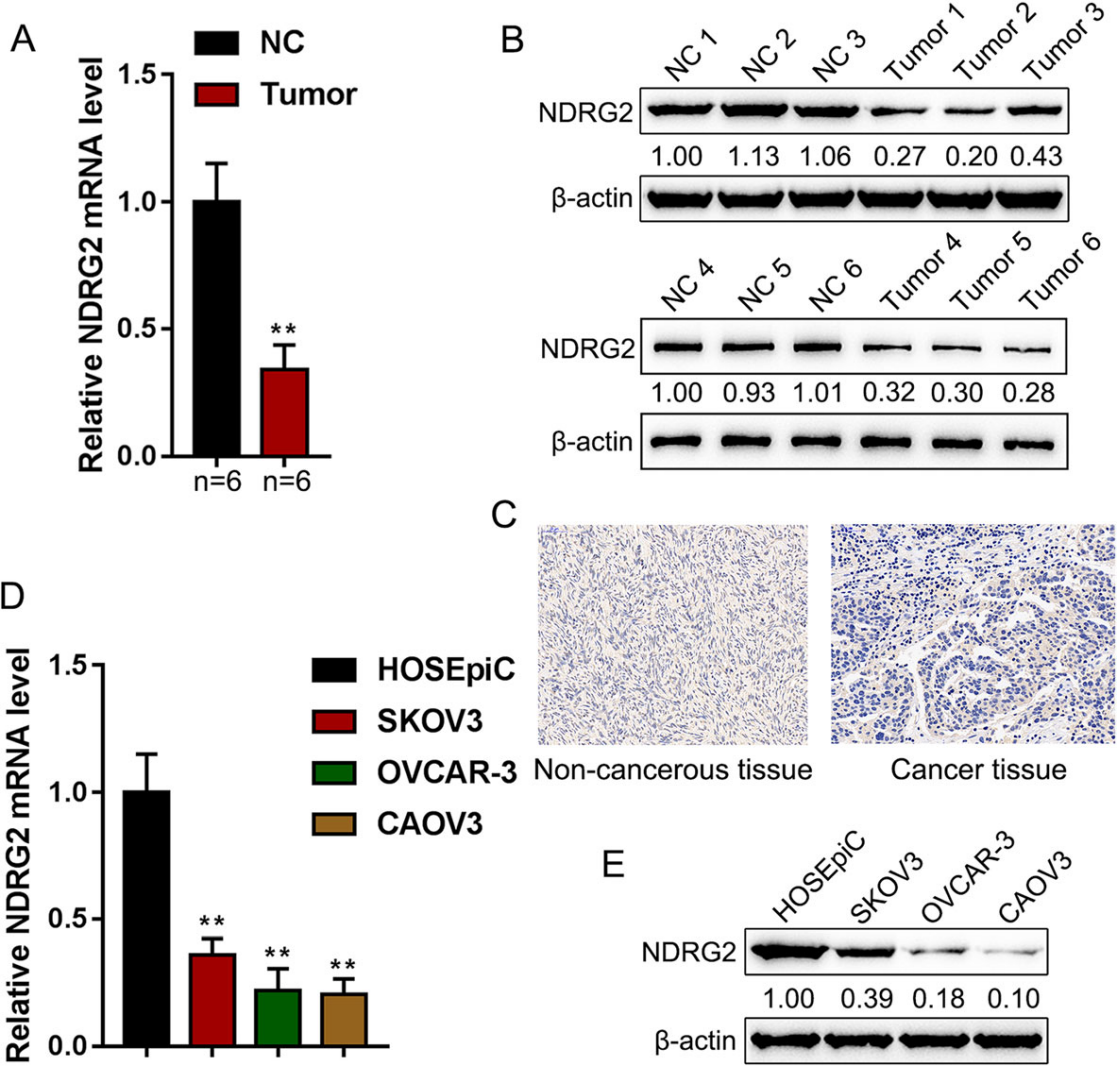
NDRG2 mRNA expression and protein levels in tissue samples and cell lines (**a** and **b**) NDRG2 mRNA and protein expression was determined in 6 paired non-cancerous and tumor tissues by real-time PCR and Immunoblotting. **c** The expressions of NDRG2 protein in non-cancerous and tumor tissues was detected by IHC assay. **d** and **e** NDRG2 mRNA and protein expression was determined in one normal cell line and three ovarian cancer cell lines, SKOV3, OVCAR-3, and CAOV3 by real-time PCR and Immunoblotting. **P < 0.01

To further confirm how NDRG2 affected ovarian cancer, we first verified NDRG2 expression within the tissues and cells of ovarian cancer. The mRNA and protein expression of NDRG2 showed to be dramatically downregulated within ovarian cancer tissues than that in non-cancerous tissue samples (Fig. 1a&b); similarly, the expressions of NDRG2 protein were lower in ovarian cancer tissues than non-cancerous tissue samples by IHC assay (Fig. 1c). Consistently, the mRNA and protein expression of NDRG2 also showed to be remarkably downregulated within three ovarian cancer cells, SKOV3, OVCAR-3, and CAOV3, than that in a normal cell line, HOSE (Fig. 1d&e).

### Involvements of NDRG2 in proliferation, apoptosis, and cell cycle of ovarian cancer cells

**Fig. 2:**
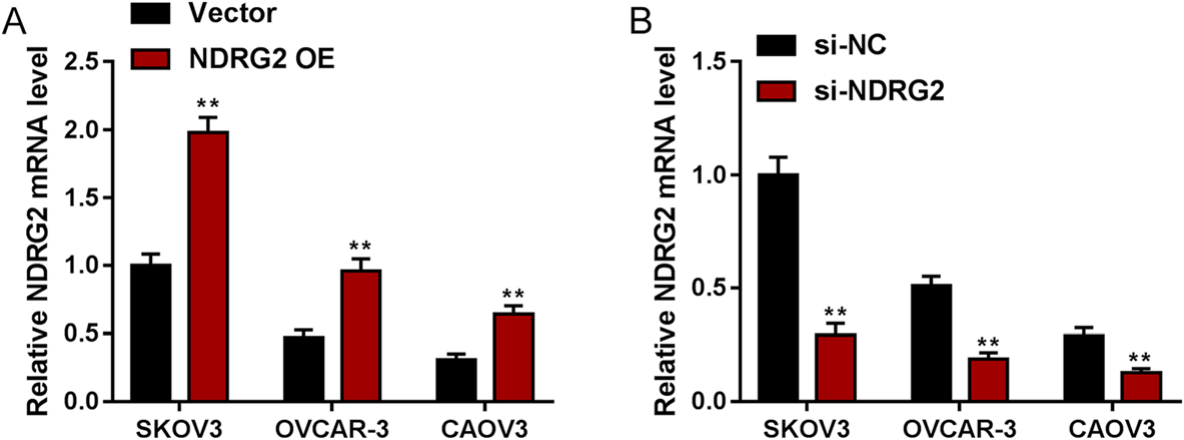
NDRG2 overexpression or silence in ovarian cancer cells (**a**) SKOV3, OVCAR-3, and CAOV3 cells were transfected with vector (negative control) or NDRG2 OE, as confirmed by real-time PCR. (**b**) SKOV3, OVCAR-3, and CAOV3 cells were transfected with si-NC (negative control) or si-NDRG2, as confirmed by real-time PCR. **P < 0.01

To further investigate the specific effects of NDRG2 on ovarian cancer cells, we conducted NDRG2 overexpression and NDRG2 silence in SKOV3, OVCAR-3, and CAOV3 cells by transfection with vector (negative control), NDRG2 OE, si-NC (negative control), or si-NDRG3. The transfection efficiency was determined via real-time PCR (Fig. 2a-b).

**Fig. 3:**
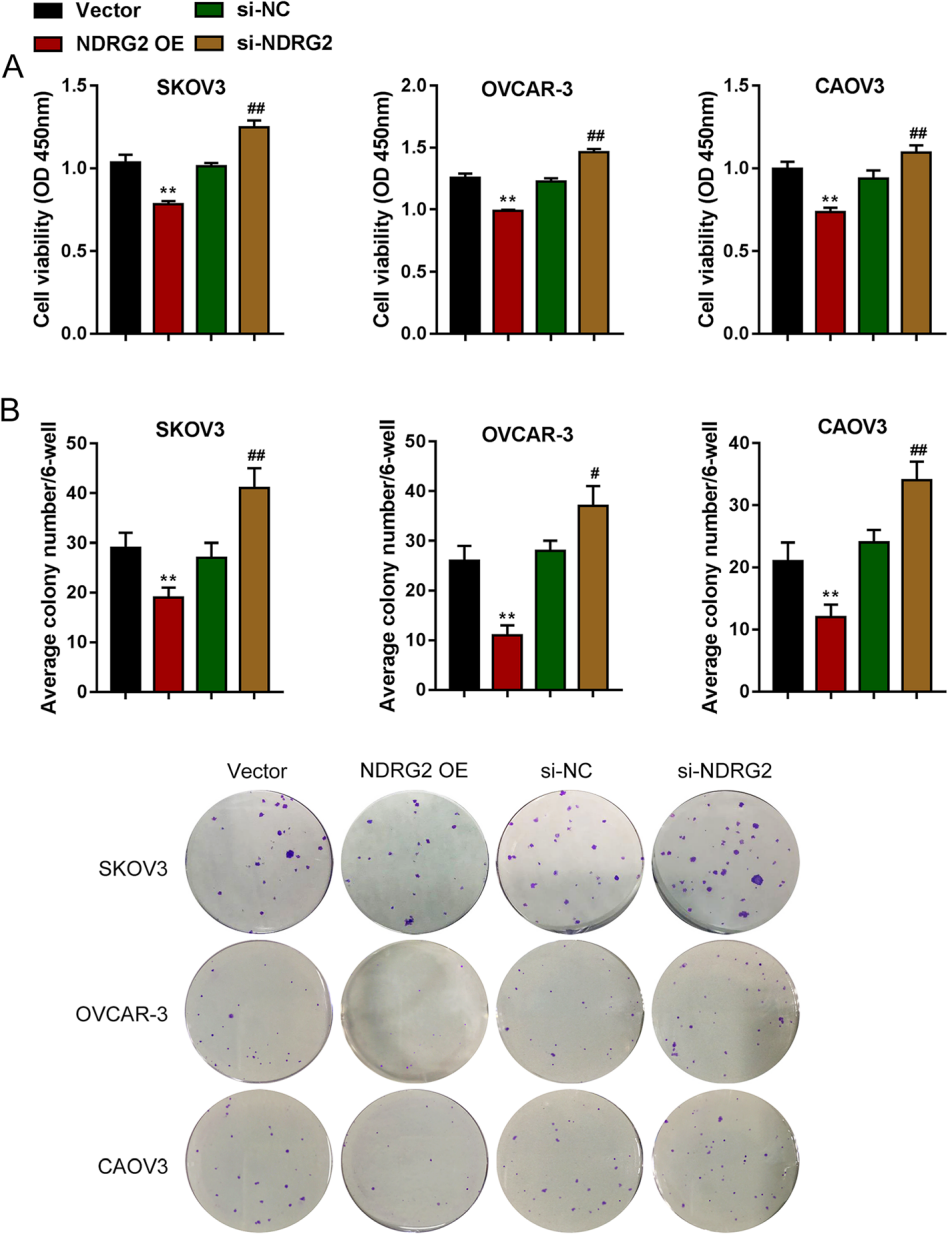
Effects of NDRG2 on ovarian cancer cell proliferation SKOV3, OVCAR-3, and CAOV3 cells were transfected with vector (negative control), NDRG2 OE, si-NC (negative control), or si-NDRG3 and examined for (**a**) cell viability by CCK-8; **b** colony formation ability. **P < 0.01, compared to Vector group; ##*P* < 0.01, compared to si-NC group

**Fig. 4:**
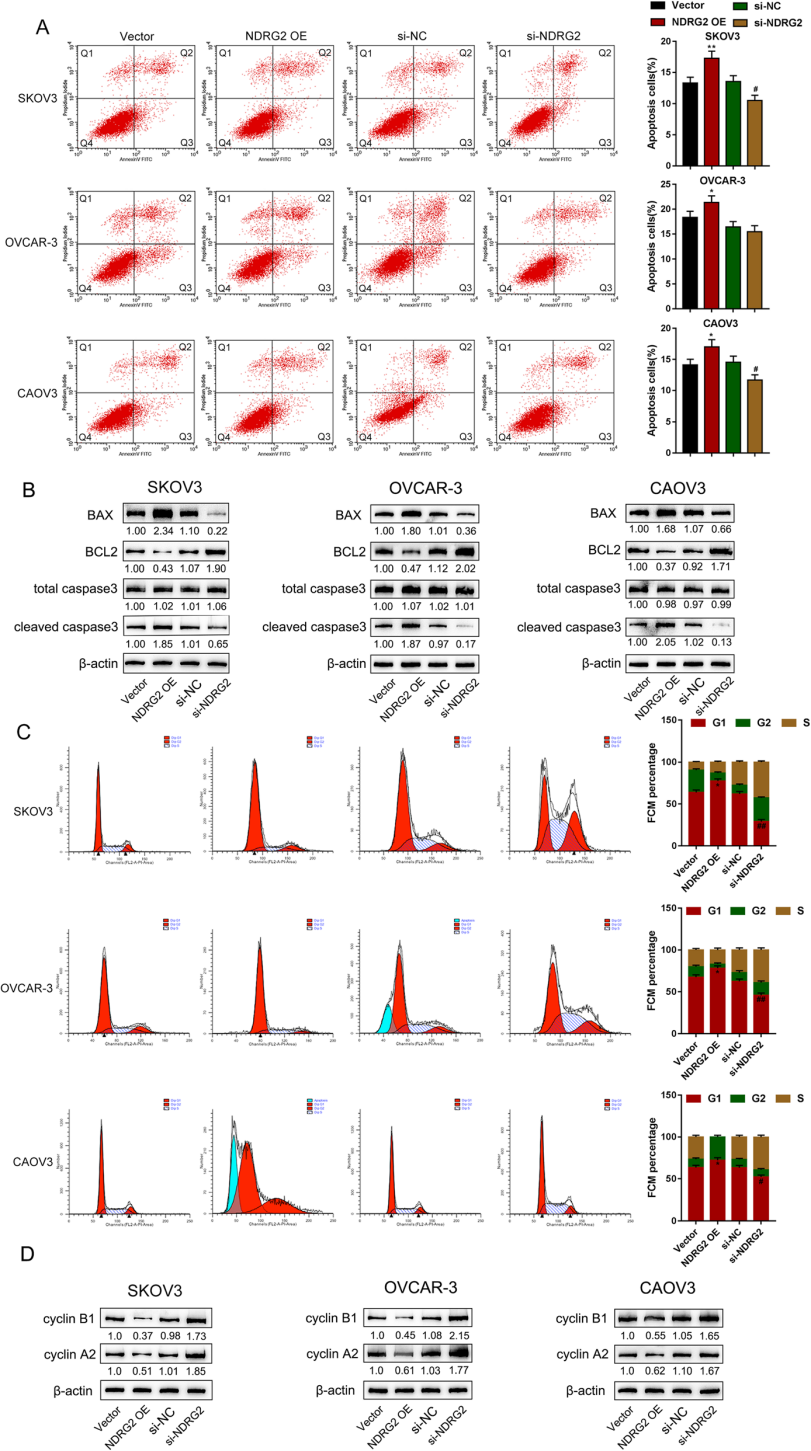
Effects of NDRG2 on ovarian cancer cell apoptosis and cell cycle SKOV3, OVCAR-3, and CAOV3 cells were transfected with vector (negative control), NDRG2 OE, si-NC (negative control), or si-NDRG3. **a** Cell apoptosis was examined by Flow cytometry. The Quadrant 1 (Q1) of the flow diagram represented as dead cells; the Quadrant 2 (Q2) represented as non-viable apoptotic cells; the Quadrant 3 (Q3) represented as viable apoptotic cells; the Quadrant 4 (Q4) represented as viable non-apoptotic cells. The percentage of cell apoptosis is the sum of Q2 and Q3 in the flow diagram. **b** Western blot assay was adopted to examine apoptosis-related protein BAX, BCL2 and cleaved caspase3/caspase3. and **c** Cell cycle was detected by Flow cytometry. **d** Cell cycle marker protein cyclin B1 and cyclin A2 had been examined by western blot assay. **P < 0.01, compared to Vector group; ##*P* < 0.01, compared to si-NC group

Next, the effects of NDRG2 overexpression and silence on ovarian cancer cells were evaluated. As revealed by CCK-8 and colony formation analyses, NDRG2 overexpression significantly suppressed, whereas NDRG2 silence promoted the cell viability and colony formation ability of SKOV3, OVCAR-3, and CAOV3 cells (Fig. 3a-b). NDRG2 overexpression significantly enhanced cell apoptosis rate to about 17.02% (SKOV3 cell), 21.37% (OVCAR-3 cell) and 17.28% (CAOV3 cell) respectively, whereas NDRG2 silence suppressed cell apoptosis rate to about 11.67% (SKOV3 cell), 15.45% (OVCAR-3 cell) and 10.51% (CAOV3 cell) respectively (Fig. 4a). Next, western blot assay was adopted to examine apoptosis-related protein BAX, BCL2 and cleaved caspase3/caspase3 in three ovarian cancer cells. NDRG2 overexpression prominently promoted, whereas NDRG2 silence inhibited apoptosis-related protein expression (Fig. 4b). Moreover, NDRG2 overexpression significantly induced the cell cycle arrested in G1 phase with increasing the percentage of G1 phase to about 77.52% (SKOV3 cell), 78.32% (OVCAR-3 cell) and 72.21% (CAOV3 cell), whereas NDRG2 silence exerted an opposing effect with reducing the percentage of G1 phase to about 29.07% (SKOV3 cell), 45.84% (OVCAR-3 cell) and 52.24% (CAOV3 cell) (Fig. 4c). Consistently, the impact of NDRG2 on cell cycle marker protein cyclin B1 and cyclin A2 had been examined by western blot assay. NDRG2 overexpression observably restrained, whereas NDRG2 silence facilitated cell cycle-related protein expression (Fig. 4d). These data indicate that NDRG2 inhibits the cell viability and colony formation, and induces apoptosis and cell cycle arrest in G1 phase, thus acting as a tumor suppressor within ovarian cancer cells.

### Effects of NDRG2 on ovarian cancer cell sensitivity to DDP treatment

**Fig. 5:**
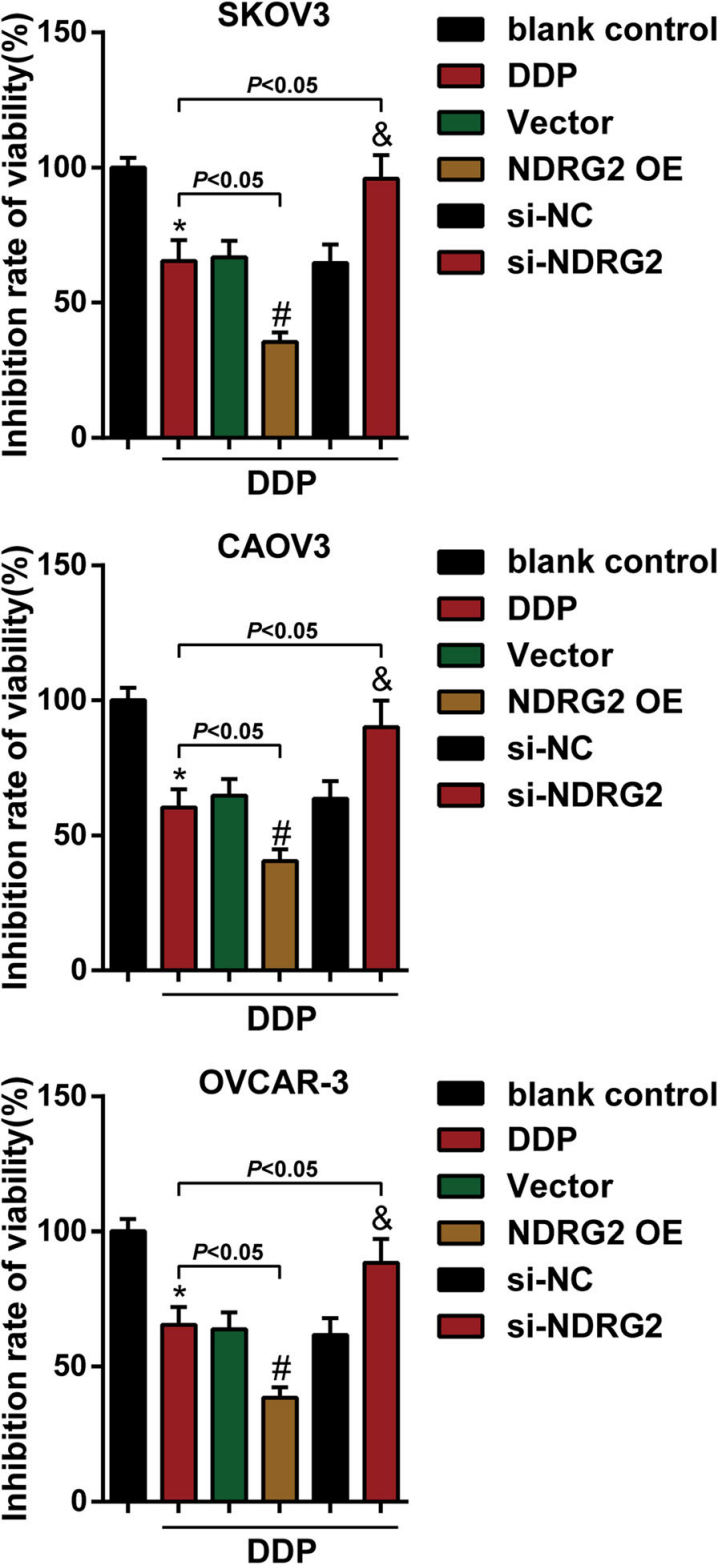
Effects of NDRG2 on ovarian cancer cell sensitivity to DDP treatment SKOV3, OVCAR-3, and CAOV3 cells were divided into blank control (untreated) group and DDP group, then cells were transfected with vector (negative control), NDRG2 OE, si-NC (negative control), or si-NDRG3 under DDP treatment and examined for cell viability by CCK-8 analysis. *P < 0.05, compared to blank control group; #*P* < 0.05, compared to Vector group; &*P* < 0.05, compared to si-NC group

To date, DDP is one of the most valid agents of ovarian cancer and other solid tumors [27–29]. After confirming the tumor-suppressive effect of NDRG2 on ovarian cancer cells, we further investigated whether NDRG2 could sensitize ovarian cancer cells to DDP treatment. Firstly, we transfected SKOV3, OVCAR-3, and CAOV3 cells with vector (negative control), NDRG2 OE, si-NC (negative control), or si-NDRG2, after 24 h, SKOV3, OVCAR-3, and CAOV3 cells were divided into blank control (untreated) group and DDP group (2.5 μg/mL DDP for 24 h) and then examined for cell viability. As shown in Fig. 5, DDP treatment dramatically inhibited cell viability; NDRG2 overexpression enhanced the suppressive role of DDP in the viability of ovarian cancer cells, whereas NDRG2 silence exerted an opposing effect. In summary, NDRG2 could improve the cellular effects of DDP on ovarian cancer cells.

### Effects of NDRG2 on the growth of xenograft formed by SKOV3 cells in nude mice

**Fig. 6:**
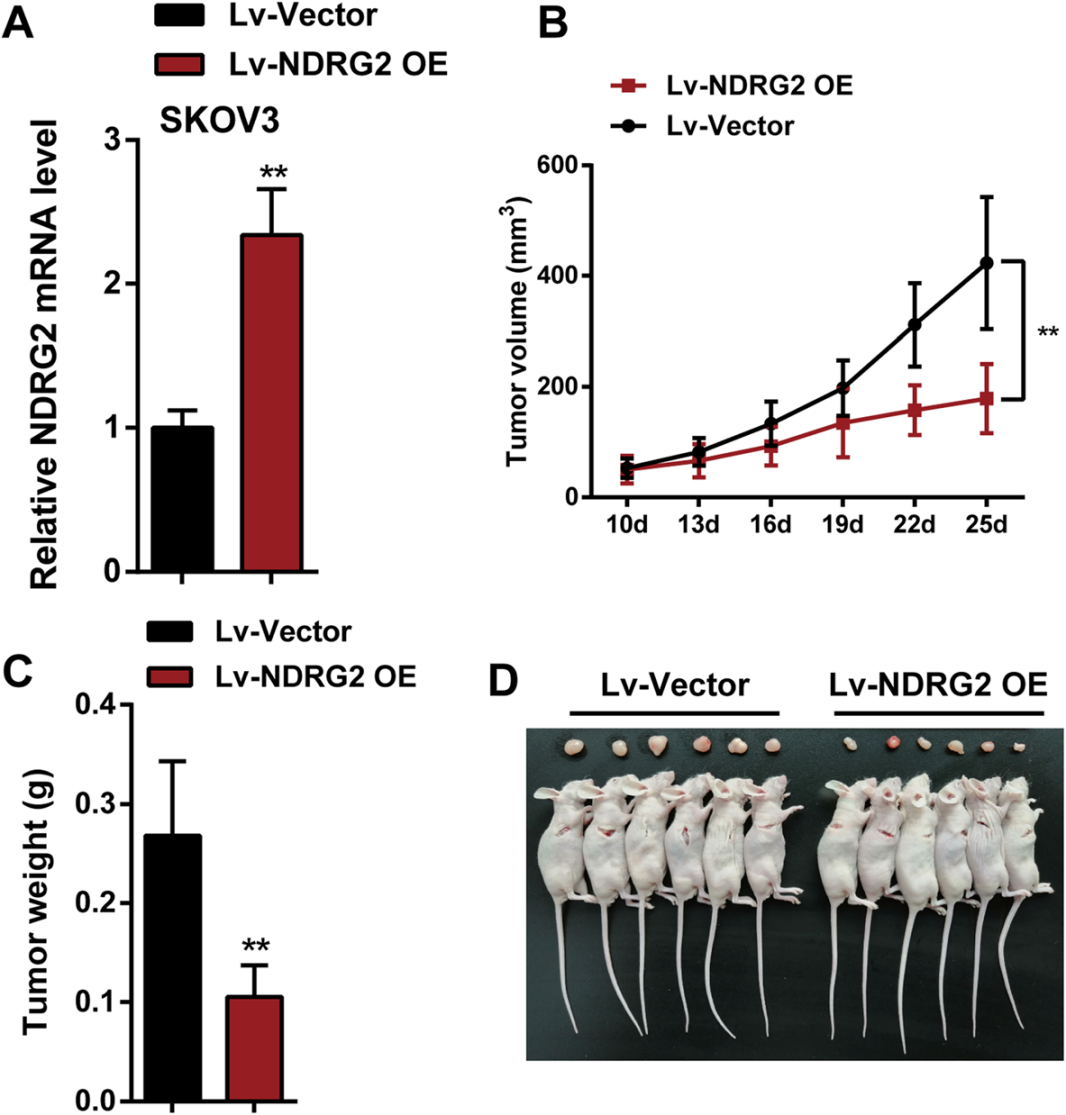
Effects of NDRG2 on the tumorigenesis of ovarian cancer in nude mice SKOV3 cells pre-transfected with lentivirus-mediated NDRG2 overexpression or negative control were subcutaneous injected into the armpit of nude mice respectively. Lv-NDRG2 OE means SKOV3 cells were transfected with lentivirus-mediated NDRG2 overexpression vector. Lv-Vector group means SKOV3 cells were transfected with noneffective negative control lentiviral vector. **a** The NDRG2 mRNA expression level in Lv-NDRG2 OE group was markedly increased compared to that in Lv-Vector group. **b** Tumor volumes of nude mice were measured every 3 days from the 10th day after injection. Overexpression of NDRG2 significantly slowed down ovarian cancer tumor growth. **c** Tumors were removed from nude mice 25 days after injection. On the 25th day, tumor weight of the Lv-NDRG2 OE group was significantly lower than that of Lv-Vector group. **d** Picture of mice tumors illustrated that overexpression of NDRG2 significantly reduced tumor size. **P < 0.01, compared to Lv-Vector group

The NDRG2 inhibition capacity towards holding the growth and tumorigenesis of ovarian cancer in vivo was evaluated on xenograft nude mice model. SKOV3 cells pre-transfected with lentivirus-mediated NDRG2 overexpression or negative control were subcutaneous injected into the armpit of nude mice respectively. The NDRG2 mRNA expression level in Lv-NDRG2 OE group was markedly increased compared to that in Lv-Vector group (Fig. 6a). During the period of xenograft growth in nude mice, measurement of tumor volumes was performed every three days. The results showed the volumes of xenograft formed by SKOV3 cells with NDRG2 overexpression were significantly less than those formed by normal SKOV3 cells since 16th day of implantation (Fig. 6b). At the end of the experiment (the 25th day), mice were euthanized and tumor tissues were excised, the weight (Fig. 6c) and sizes (Fig. 6d) of xenograft formed by SKOV3 cells with NDRG2 overexpression were clearly smaller than those formed by normal SKOV3 cells. Therefore, NDRG2 overexpression attenuated the growth and tumorigenesis of ovarian cancer in vivo.

## Discussion

Herein, we revealed that NDRG2 mRNA expression and protein levels were downregulated within both ovarian cancer tissues and cell lines. The overexpression of NDRG2 dramatically inhibited the cell viability, colony formation and cell cycle marker protein cyclin B1 and cyclin A2 expression levels, whereas promoted the cell apoptosis and apoptosis-related protein BAX, BCL2 and cleaved caspase3/caspase3 expression levels and cell cycle arrest in G1 phase within ovarian cancer cells. More importantly, NDRG2 overexpression significantly enhanced the suppressive roles of DDP in ovarian cancer cell viability. NDRG2 overexpression attenuated the growth and tumorigenesis of ovarian cancer in vivo. On the contrary, NDRG2 silence exerted opposing effects on ovarian cancer cells.

NDRG2 is one member of the NDRG family that contains NDRG1, NDRG2, NDRG3, and NDRG4 [30, 31]. NDRG2 has been reported to be reduced within colorectal cancer, liver cancer, thyroid cancer, glioblastoma, breast cancer and a number of other human cancers [16, 17, 20, 23, 32, 33]. Moreover, the increase in NDRG2 expression within cancer cells can be related to improved prognosis in gastric cancer, high-grade glioma and hepatocellular carcinomas [17, 34, 35]. Herein, the mRNA and protein expression of NDRG2 showed to be dramatically downregulated within the ovarian cancer tissues than that in the normal controls. In vitro, the mRNA and protein expression of NDRG2 also showed to be remarkably downregulated within SKOV3, OVCAR-3, and CAOV3 cells than those within a non-cancerous ovarian epithelial cell line, HOSE. Since excessive NDRG2 protein within cancer cells leads to significantly decreased cell proliferation [16, 36, 37] while the increased mRNA expression of NDRG2 can be related to improved prognosis [17, 34], we speculate that NDRG2 might act as a tumor suppressor within ovarian cancer, possibly by regulating ovarian cancer cell proliferation and apoptosis.

To verify the above-mentioned speculation, we conducted NDRG2 overexpression or silence in ovarian cancer cells and evaluated its cellar effects. Consistent with its expression pattern, NDRG2 acts as a tumor suppressor within ovarian cancer cell lines. NDRG2 overexpression dramatically suppressed the proliferation whereas enhanced the apoptosis of ovarian cancer cells, similarly to its effects on other cancers [22, 38–40]. Also, the effects of NDRG2 on the growth of xenograft formed by SKOV3 cells in nude mice had been investigated in the study. NDRG2 overexpression attenuated the growth and tumorigenesis of ovarian cancer in vivo. Besides, the resetting of the G1, S, G2, M phases and other cell cycles can be found in the occurrence and development of tumors. As confirmed by a GO (Gene Ontology) enrichment analysis on the biological process, molecular function and cellular component, the overexpression of NDRG2 can increase the G protein signaling-associated genes while reduce the M phase-associated gene sets, which is consistent with cell cycle analyses [41]. Analyses on the signaling pathways have also revealed the decreased glycosylphosphatidylinositol (GPI)-anchor biosynthesis and protein degradation [41]. It has also been demonstrated by Ma et al. [19] that G1 arrest can be induced by the expression of NDRG2. NDRG2 was introduced into SW620 cells, after which the cell cycle arrest was observed to arrest at G1/S phase [37]. The expression of NDRG2 can effectively inhibit cell cycle resetting within tumors. Herein, we observed similar results that, NDRG2 overexpression dramatically induced the cell cycle arrest in G1 phase and inhibited cell cycle marker protein cyclin B1 and cyclin A2 expression levels in all the three ovarian cancer cell lines, suggesting that NDRG2 might suppress ovarian cancer cell proliferation via affecting the resetting of cancer cell cycle.

Cisplatin is the most common platinum-based chemotherapy drug. Its action mechanism is DNA cross-linking, so its tumor-suppressive activity is broad-spectrum and non-cell cycle-specific, leading to the inhibition of DNA replication and transcription and induction of tumor cell apoptosis [42, 43]. Although cisplatin has been considered an effective agent for ovarian cancer treatment, the acquisition and development of drug resistance has emerged as a primary obstacle to its wide clinical application [44]. Previously, Liu et al. [45] reported that NDRG2 silence inhibits the expression of Bcl-2, so that cervical cancer Hela cells can be sensitive to cisplatin. In histiocytic lymphoma U937 cells, NDRG2 could modulate NOX5-ROS-PKR pathway-regulated Bak-to-Mcl-1 ratio to increase the sensitivity to cisplatin [46]. In the present study, NDRG2 overexpression within ovarian cancer cell lines significantly enhanced the suppressive effects of DDP upon the viability of ovarian cancer cells, whereas NDRG2 silence exerted an opposing effect. These data indicate that NDRG2 might sensitize ovarian cancer cells to DDP treatment.

## Conclusion

Taken together, our findings provide a solid experimental basis demonstrating the cellular effects of NDRG2 in inhibiting the cell proliferation, enhancing the cell apoptosis, eliciting the cell cycle arrest in G1 phase, and promoting the suppressive effects of DDP upon the viability of cancer cells. NDRG2 overexpression attenuated the growth and tumorigenesis of ovarian cancer in vivo. Nevertheless, it is still necessary to further study the new strategy of rescuing the abnormally downregulated NDRG2 expression as a promising therapeutic strategy for clinical applications.

## Data Availability

All data generated or analyzed during this study are included in this published article.
